# Oral Health Screening Prior to Oncological Treatment—The Helsinki Protocol

**DOI:** 10.1111/odi.70339

**Published:** 2026-04-08

**Authors:** Jemina Järvinen, Johanna Uittamo, Hanna Välimaa, Tero Puolakkainen, Aleksi Haapanen

**Affiliations:** ^1^ Department of Oral and Maxillofacial Diseases University of Helsinki Helsinki Finland; ^2^ Department of Oral and Maxillofacial Diseases Helsinki University Hospital Helsinki Finland; ^3^ Meilahti Vaccine Research Centre MeVac, Department of Infectious Diseases University of Helsinki and Helsinki University Hospital Helsinki Finland

**Keywords:** head and neck cancer, medication‐related osteonecrosis of the jaw, multiple myeloma, oral health, osteoradionecrosis

## Abstract

**Objectives:**

Oncological treatments can have adverse effects on oral health and cause long‐term complications such as osteonecrosis. This retrospective study investigated the oral health of head and neck cancer patients before radiotherapy and patients with multiple myeloma, breast, prostate, lung, and kidney cancers before antiresorptive therapy.

**Materials and Methods:**

Altogether 154 patients referred to dental screening before initiation of these therapies at Helsinki University Hospital in 2021 were included. Under the Helsinki protocol, the aim was to assess and treat the patients in a single day, progressing from examination to counseling and, if needed, to extractions.

**Results:**

Before oncological treatment, 61.7% of the patients required at least one tooth extraction due to infection foci. Among antiresorptive therapy patients, multiple myeloma patients had less need for extraction (*p* = 0.006). Head and neck cancer patients smoked and used alcohol more than antiresorptive therapy patients. Smoking was associated with the need for extractions (*p* = 0.001).

**Conclusion:**

The Helsinki protocol enables efficient oral health evaluation without delaying cancer treatment. Head and neck cancer patients and antiresorptive therapy patients need dental evaluation before oncological treatment. Over half of patients had infection foci requiring extraction. Multiple myeloma patients showed a lower extraction need than other antiresorptive therapy patients.

## 1 Introduction

The necessity of eliminating dental foci before initiating oncological therapy remains debated (Kashiwazaki et al. 2012; Hong et al. 2018; Spijkervet et al. 2021). Although acute dental infections during cancer treatment appear relatively uncommon, with a reported prevalence of 5.4% (Hong et al. 2018), many oral infections—particularly periodontal disease—are chronic and require multiple dental visits. Because cancer treatment must typically commence without delay, the time available for comprehensive oral management is limited. Radiotherapy, chemotherapy, and antiresorptive drugs, especially in patients with multiple myeloma or bone metastases, all have the potential to adversely affect oral health, underscoring the importance of timely dental evaluation prior to oncologic care (Spijkervet et al. 2021; Rugani et al. 2016).

In head and neck cancer (HNC), oral health must be assessed prior to oncological treatment, as radiotherapy (RT) or chemoradiotherapy (CRT) is commonly used alongside surgical care. RT/CRT adversely affects oral health by increasing infection risk, reducing salivary flow, and altering saliva composition, and predisposing to caries, periodontal disease, mucositis, and osteoradionecrosis (ORN) (Sroussi et al. 2017). Salivary gland damage leads to hyposalivation (Möller et al. 2004) and an elevated long‐term caries risk (Jham et al. 2008; Moore et al. 2020). Consequently, dental examination, necessary treatment, and planning of post‐treatment oral care should be completed before RT/CRT, accompanied by appropriate oral health counseling. Most RT patients develop clinically significant mucositis, causing pain, ulceration, and bleeding, which impairs quality of life (Duncan et al. 2005; Trotti et al. 2003; Kim et al. 2012).

Dental extractions increase the risk of ORN in RT/CRT patients (Reuther et al. 2003), and pre‐existing dental infections, including periodontitis, further contribute to osteonecrosis risk (Katsura et al. 2008). Teeth with poor prognosis may therefore require removal before RT. ORN can occur in both jaws but is more common in the mandible, with reported incidence ranging from 2% to 8.2% after RT (Reuther et al. 2003; Nabil and Samman 2012). The International Society of Oral Oncology (ISOO), the Multinational Association for Supportive Care in Cancer (MASCC), and the American Society of Clinical Oncology (ASCO) have published guidelines addressing the prevention and management of ORN in these patients (Peterson et al. 2024). According to the ISOO‐MASCC‐ASCO guidelines, any necessary tooth extractions should be performed at least two weeks prior to RT. After RT, patients who have received a radiation dose of 50 Gy or higher to the jaw should maintain good oral hygiene, and any dentoalveolar surgical procedures should be carefully considered (Peterson et al. 2024). Guidance on managing hyposalivation is also essential. Ensuring that patients are fully informed about the oral health consequences of their cancer treatment remains a critical component of care (Carvalho et al. 2018; Jané‐Salas et al. 2022).

Antiresorptive agents such as bisphosphonates and denosumab increase the risk of medication‐related osteonecrosis of the jaw (MRONJ) (Jadu et al. 2007; Drake et al. 2008). These drugs are widely used in osteoporosis, metabolic bone disorders, and cancer‐related conditions, particularly in patients with multiple myeloma or solid tumors with bone metastases, most commonly breast and prostate cancers (Drake et al. 2008). Although MRONJ may arise spontaneously, it most often follows dental extraction (Jadu et al. 2007; McGowan et al. 2019), and can also be triggered by acute or chronic oral infection or dental trauma (McGowan et al. 2019). Long‐term high‐dose therapy further elevates the risk (Jadu et al. 2007; Wick et al. 2022; Bamias et al. 2005). Reported MRONJ prevalence is approximately 2% in breast cancer, 3.8% in prostate cancer, and 5% in multiple myeloma patients receiving antiresorptive treatment (Rugani et al. 2016). Similar to ORN, MRONJ predominantly affects the mandible (Jadu et al. 2007).

The aim of this study was to investigate the oral health of head and neck cancer patients prior to RT/CRT, as well as that of patients with multiple myeloma or breast, prostate, lung, or kidney cancer with bone metastases before initiation of antiresorptive therapy. We hypothesized that most patients would present with oral infection foci necessitating tooth extraction prior to oncologic treatment. The study further sought to determine the need for dental interventions and to assess differences between patient groups, thereby clarifying the overall necessity of dental care before cancer therapy.

## 2 Materials and Methods

### 2.1 Study Design

A retrospective study was conducted to investigate the oral findings and need for dental treatment of patients diagnosed with HNC or malignancy requiring initiation of antiresorptive therapy, who were referred to dental screening at Helsinki University Hospital from January 2021 to December 2021.

### 2.2 Inclusion Criteria

Patients aged ≥ 18 years who had been referred to dental screening at Helsinki University Hospital prior to initiation of antiresorptive drug treatment for a malignancy or head and neck radiation therapy (single modality), or chemoradiotherapy were included in the study. Individuals younger than 18 years old and patients whose oncologic management consisted solely of cytostatic agents or surgery were excluded from the study.

### 2.3 The Helsinki Protocol

At Helsinki University Hospital, a dental infection focus screening is performed routinely before head and neck RT/CRT or initiation of antiresorptive drug treatment for cancer patients. The purpose of the infection focus examination is to evaluate the oral health status and self‐care routines of the patient and to find and treat potential acute and chronic dental infections that would affect the timing and execution of upcoming oncologic treatment. Additionally, focus screening allows counseling to be given prior to oncological treatment. Since 2020, patients in our clinic have been examined and treated according to the Helsinki protocol, i.e., a multi‐room model (Figure 1).

**FIGURE 1:**
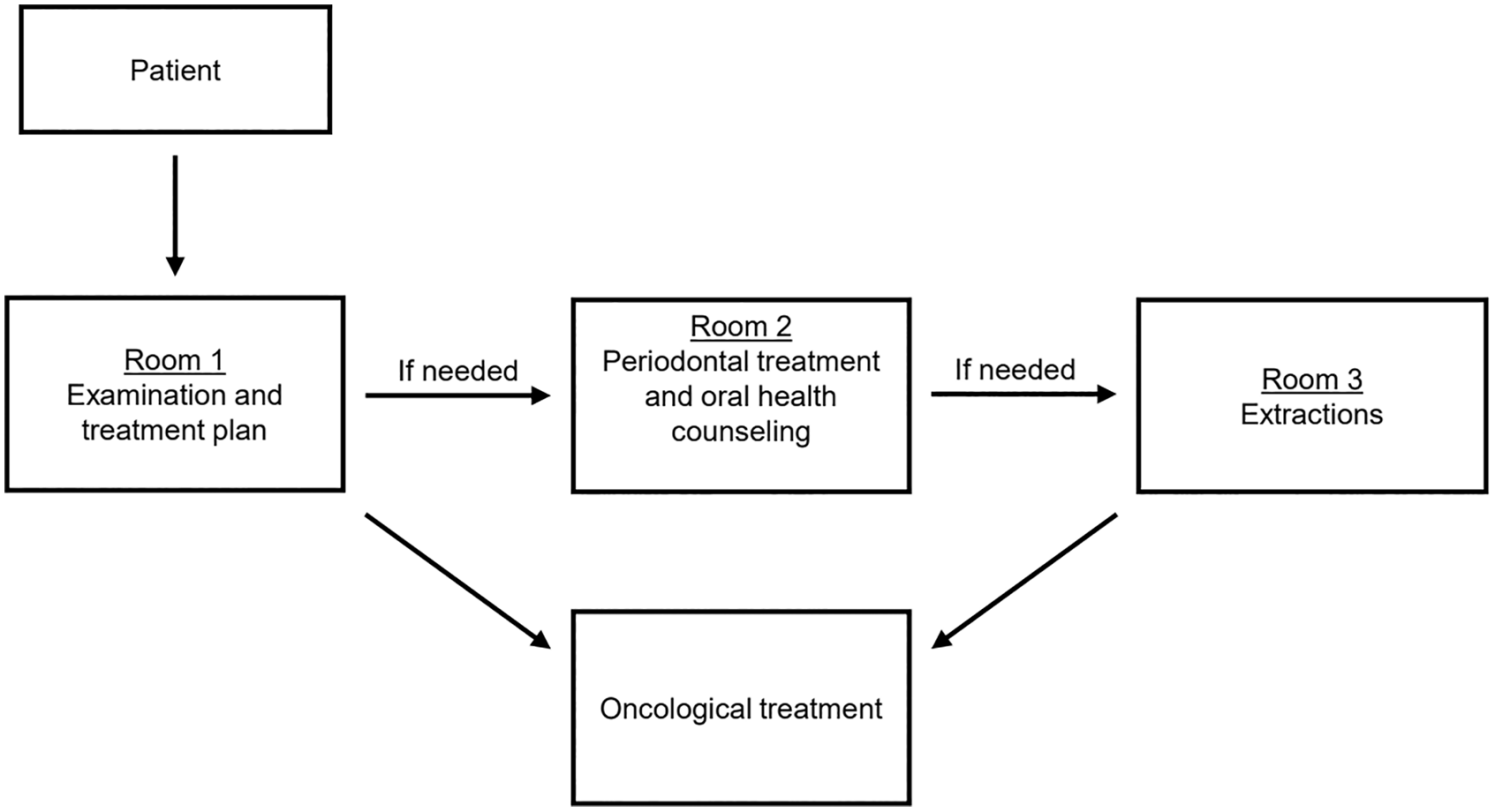
The Helsinki protocol.

Briefly, in the first room, the patient is examined, and a treatment plan is devised by a specialized dentist or a resident in the field of clinical dentistry. A dental panoramic radiograph (DPR) was obtained for all patients prior to the dental examination. An individualized treatment plan is developed for each patient, considering the reason for referral and the schedule of oncological treatment. In identifying potential infection foci, we follow Finnish Current Care Guidelines, which align with protocols such as the one presented by Yamagata et al. (Yamagata et al. 2006). A more detailed description of the potential infection foci is provided in the Study variables section below. In the second room, conservative periodontal treatment is performed, and oral health counseling is given by a dental hygienist. Finally, in the third room, tooth extractions are performed by an oral and maxillofacial surgical resident. The goal of this multi‐room model is to eliminate clinically relevant oral infectious foci in a single visit to reduce oral health‐associated complications in cancer patients and to allow oncological treatment to begin without delay. Before the dental examination, a DPR and necessary blood samples are taken from the patient. Preoperative DPR images were interpreted by an oral radiologist. Intraoral x‐rays may be taken during the examination as needed. Root canal treatments and fillings are seldom provided due to the limited time frame before oncological treatments. This multi‐room model is seen as effective and equal for all patients. It also serves as an educational rotation for specializing dentists.

### 2.4 Study Variables

The primary outcome was the need for tooth extraction. Study variables included type of malignancy (HNC, multiple myeloma and breast, prostate, lung, and kidney cancers with bone metastases), type of cancer treatment (RT/CRT and antiresorptive therapy), age, sex, time from referral to appointment (days), and history of smoking and alcohol use. Moderate alcohol use according to the Finnish Current Care Guidelines is ≥ 14 doses of alcohol per week for men and ≥ 7 doses for women (Working group appointed by the Finnish Medical Society Duodecim and the Finnish Society of Addiction Medicine 2018). One dose contains 12 g of pure alcohol (Working group appointed by the Finnish Medical Society Duodecim and the Finnish Society of Addiction Medicine 2018). Potential dental foci included deep caries lesions extending close to the pulp with a potential of causing infection of the root canal, periapical periodontitis, residual roots, partially erupted teeth associated with swelling and purulent discharge, teeth with at least 6 mm periodontal pockets, class III furcation (Glickman Classification), and grade III mobility (Miller Classification). Information on apical periodontitis, unerupted teeth, and vertical periodontal bone loss was collected from the radiologists' reports. A treatment plan was made specifically based on patients' oral health, prognosis of the underlying disease, and self‐care habits. Overall, teeth with symptomatic deep caries or periapical periodontitis, symptomatic pericoronitis, acute infection, or grade III mobility were classified as indications for extraction.

### 2.5 Statistical Analysis

Descriptive statistics are presented as absolute values and percentages. Pearson's Chi‐square test was used for categorical variables when all expected cell frequencies were ≥ 5. Fisher's Exact test was applied when any expected cell count was < 5. Continuous variables that were not normally distributed were analyzed using the Mann–Whitney *U* test. Statistical significance was set at *p* < 0.05. Statistical analyses were performed using SPSS version 25.0 (IBM Corp., Armonk, NY, USA).

## 3 Results

Altogether 154 patients were included in the study of which 93 (60.4%) were men and 61 (39.6%) women. Age ranged from 43 to 93 years; the median age was 69 years. A total of 87 patients were about to receive head and neck RT/CRT and 67 patients were on antiresorptive drug treatment (34 for multiple myeloma, 16 for breast cancer, 11 for prostate cancer, and six for other malignancies (kidney and lung cancer)). Of all patients, 61.7% had at least one tooth that had to be extracted before oncological treatment (Table 1). Also, 79.9% of patients required periodontal treatment prior to oncological treatment. Overall, 59.7% of all patients completed the proposed treatment in a single day. For the others, the treatment was completed within a few days of the examination. Reasons for some patients not completing the treatment on the same day included time constraints, unwillingness to undergo treatment that day, or ineligibility for treatment. None of the patients' oncological treatment was delayed due to dental treatment and the average interval between tooth extraction and the initiation of cancer treatment was approximately 2–4 weeks.

**TABLE 1:** Descriptive statistics of 154 patients.

	*n* = 154	%
*Age*		
Range	43–93	
Mean	68.2	
Median	69	
*Sex*		
Male	93	60.4
Female	61	39.6
*Smoking status*		
Current smoker/former smoker	73	47.4
Never‐smoker	63	40.9
Status unknown	18	11.7
*Moderate alcohol use*		
Yes	18	11.7
No	96	62.3
Status unknown	40	26.0
*Timespan from referral to appointment (days)*		
Range	1–97	
Mean	14.3	
Median	13	
*Reason for referral*		
Head and neck malignancy	87	56.5
Breast cancer	16	10.4
Prostate cancer	11	7.1
Myeloma	34	22.1
Other	6	3.9
*Need for teeth extractions*		
Yes	95	61.7
No	59	38.3
*Number of extracted teeth*		
0	59	38.3
1–3	55	35.7
4–6	21	13.6
Over 6	19	12.3

HNC patients were predominantly male (*p* < 0.001) and had more alcohol use (*p* = 0.003) and smoking (*p* = 0.006) than patients receiving antiresorptive therapy. No significant difference was noted in the need for tooth removal between HNC patients and antiresorptive therapy patients. Of the HNC patients, 42.5% had periapical periodontitis, 36.8% had periodontal disease (pocket depth > 5 mm), 12.6% had deep caries lesions, and 64.4% needed tooth extraction. The result is similar for the antiresorptive therapy patients, the corresponding figures being 43.3%, 29.9%, 11.9%, and 58.2% (Table 2). However, within the group of antiresorptive therapy patients, those with myeloma had less need for dental extractions than the other antiresorptive therapy patients (no need for dental extractions in 61.8% of the myeloma patients vs. 21.2% of the other antiresorptive drug patients, *p* = 0.006) (Table 3). The reasons for tooth extraction were deep caries, severe periodontitis, residual roots, and partially erupted teeth associated with pericoronitis, swelling or purulent discharge.

**TABLE 2:** Comparison of descriptive statistics according to each disease group.

	HNC patients (*n* = 87)	% of HNC patients	Antiresorptive therapy patients (*n* = 67)	% of antiresorptive therapy patients	*p*
*Sex*					
Male	63	72.4	30	44.8	< 0.001
Female	24	27.6	37	55.2	
*Age*					
Range	43–89		46–93		
Mean	67.1		69.6		0.078
Median	68		72		
*Moderate alcohol use*					0.003
Yes	17	19.5	1	1.5	
No	51	58.6	45	67.2	
Status unknown	19	21.8	21	31.3	
*Smoking status*					0.006
Current smoker/former smoker	52	59.8	21	31.3	
Never‐smoker	28	32.2	35	52.2	
Status unknown	7	8.0	11	16.4	
*Dental status*					
Periapical periodontitis	37	42.5	29	43.3	0.925
> 5 mm pocket	32	36.8	20	29.9	0.367
Vertical periodontal bone loss	24	27.6	18	26.9	0.921
Acute infection	17	19.5	8	11.9	0.205
Residual root	14	16.1	16	23.9	0.226
Deep caries lesions	11	12.6	8	11.9	0.895
Unerupted teeth	9	10.3	4	6.0	0.333
Class III furcation	8	9.2	4	6.0	0.459
Grade III mobility	8	9.2	3	4.5	0.351*
*Number of extracted teeth*					0.393
0	31	35.6	28	41.8	
1–3	32	36.8	23	34.3	
4–6	15	17.2	6	9.0	
Over 6	9	10.3	10	14.9	

*Note:* Dental status percentages may exceed 100% because multiple dental findings could be recorded for the same patient. *p*‐values marked with an asterisk (*) were calculated using Fisher's exact test.

**TABLE 3:** Comparison between different antiresorptive patient groups.

	Multiple myeloma patients (*n* = 34)	% of	Other malignancies (*n* = 33)	% of	*p*
*Sex*					
Male	15	44.1	15	45.5	0.912
Female	19	55.9	18	54.5	
*Age*					
Range	46–84		48–93		0.656
Mean	69.2		69.3		
Median	71.5		72		
*Moderate alcohol use*					0.406
Yes	0	0.0	1	3.0	
No	22	64.7	23	69.7	
Status unknown	12	35.3	9	27.3	
*Smoking status*					0.264
Current smoker/former smoker	8	23.5	13	39.4	
Never‐smoker	18	52.9	17	51.5	
Status unknown	8	23.5	3	9.1	
*Dental status*					
Periapical periodontitis	11	32.4	18	54.5	0.067
> 5 mm pocket	5	14.7	15	45.5	0.006
Vertical periodontal bone loss	7	20.6	11	33.3	0.239
Acute infection	1	2.9	7	21.2	0.027*
Residual root	4	11.8	12	36.4	0.023
Deep caries leesions	1	2.9	7	21.2	0.027
Unerupted teeth	2	5.9	2	6.1	1*
Class III furcation	2	5.9	2	6.1	1*
Grade III mobility	1	2.9	2	6.1	0.614*
*Number of extracted teeth*					0.006*
0	21	61.8	7	21.2	
1–3	9	26.5	14	42.4	
4–6	2	5.9	4	12.1	
Over 6	2	5.9	8	24.2	

*Note:* Dental status percentages may exceed 100% because multiple dental findings could be recorded for the same patient. *p*‐values marked with an asterisk (*) were calculated using Fisher's exact test.

Table 4 shows that smoking status showed a significant unadjusted association with the need for dental extractions, with extractions being more common among current and former smokers than among never‐smokers (*p* = 0.001).

**TABLE 4:** Association of need for tooth extraction with variables.

	Need for tooth extraction (*n* = 95)	% of	No need for tooth extraction (*n* = 59)	% of	*p*
All	95	61.69	59	38.31	
*Age*					
Range	47–89		43–86		
Mean	69.3		66.3		0.104
Median	69		68		
*Sex*					
Male	60	64.5	33	35.5	0.373
Female	35	57.4	26	43.6	
*Smoking status*					0.001
Current smoker/former smoker	56	76.7	17	23.3	
Never‐smoker	29	46.0	34	54.0	
Status unknown	10	55.6	8	44.4	
*Moderate alcohol use*					0.413
Yes	13	76.5	4	23.5	
No	57	60.0	38	40.0	
Status unknown	25	59.5	17	40.5	
Timespan from referral to appointment (days) median	13.7		15.3		0.626
Reason for referral					
Head and neck cancer	56	64.4	31	35.6	0.436
Antiresorptive therapy patients	39	58.2	28	41.8	
Multiple myeloma	13	38.2	21	61.8	0.001
Other malignancies	26	78.8	7	21.2	

## 4 Discussion

The current study revealed that a substantial proportion of patients were found to require dental intervention prior to oncological treatment, highlighting the clinical significance of pre‐treatment oral assessment. Although the need for oral screening before cancer therapy is well recognized (Hong et al. 2018; Spijkervet et al. 2021), there is currently no consensus regarding dental treatment protocols or which dental conditions require urgent management. Nevertheless, evidence indicates that acute and symptomatic oral infections increase the risk of complications such as MRONJ (Jadu et al. 2007; McGowan et al. 2019), supporting the rationale for eliminating potential dental infection foci before oncologic treatment.

The aim of the current study was to clarify the oral health status of different cancer patient groups prior to head and neck cancer (HNC) radiotherapy or chemoradiotherapy (RT/CRT) or antiresorptive drug therapy. Our hypothesis was confirmed, as 61.7% of patients receiving HNC or antiresorptive treatment required tooth extraction before the initiation of oncological therapy (Table 1). Previous studies have reported that approximately half of HNC patients require at least one tooth extraction prior to radiotherapy (Jham et al. 2008). Tooth extraction has consistently been identified as the most common dental procedure performed before cancer treatment, followed by restorative procedures and professional dental cleaning (Bertl et al. 2022). These findings further emphasize the importance of comprehensive clinical and radiological dental evaluation in this patient population.

In our study, the main diagnoses were periapical periodontitis, periodontal disease, and caries, and more than 60% of patients needed tooth extraction. Similar results were reported in a study conducted in Brazil, which showed that 41.0% of HNC patients had periodontal disease, 12% had caries lesions, and 50.2% needed at least one tooth extracted (Jham et al. 2008).

Our results reveal no significant differences in tooth extraction needs between HNC patients and antiresorptive therapy patients (Table 2). However, myeloma patients tend to have a lower need for tooth extraction compared with other patients receiving antiresorptive therapy. Differences were also observed in dental status between these groups (Table 3). All categories of dental findings were less frequent among multiple myeloma patients than among other antiresorptive therapy patients. For example, acute infections were observed in only 2.9% of multiple myeloma patients, compared with 21.2% of patients receiving other antiresorptive therapies. Among patients receiving other antiresorptive therapies, 78.8% required tooth extractions prior to oncological treatment, whereas among patients with multiple myeloma, only 38.2% required extractions (Table 3). Previous studies have shown that hematopoietic stem cell transplant patients also need dental treatment (Skallsjö et al. 2023).

The purpose of the Helsinki protocol is to examine and treat patients on the same day, allowing oncological treatment to start promptly. Of the patients included in this study, 59.7% completed the proposed treatment in a single day. Patients did not complete the treatment on the same day due to factors such as limited available time, unwillingness to undergo treatment that day, or ineligibility for treatment. In addition to extractions, patients with gingivitis or periodontitis also visited a dental hygienist for periodontal treatment and oral health counseling. Overall, 79.9% of the patients required periodontal treatment prior to oncological treatment.

Oncological patients have a need for dental treatment and would benefit from oral health evaluation prior to oncological treatment. Earlier studies have shown that patients treated by a team without a dental professional are at higher risk of not consulting a dentist at all after cancer diagnosis and prior to oncological treatment (Bertl et al. 2016). However, if HNC patients have dental professionals included in their multidisciplinary team, their oral health is better 6 months and longer after finishing cancer therapy (Bertl et al. 2022).

Alcohol drinking and smoking are associated with an increased risk of oral cancer (Gupta et al. 2017; Bagnardi et al. 2015). Our study shows that HNC patients use more alcohol and tobacco products than antiresorptive therapy patients (Table 2). Among antiresorptive therapy patients, individuals with multiple myeloma had the lowest rates of alcohol and tobacco use (Table 3). Smoking has been widely associated with impaired oral health (Tanner et al. 2015), and in the present study, smoking showed a significant unadjusted association with the need for dental extractions (Table 4). These findings suggest that extra attention should be given to HNC patients with a known history of substance abuse and smoking, and the oral health of these patients should be evaluated before oncological treatment.

An advantage of this study is that dental examinations were conducted by a limited number of dentists using a similar protocol in a single center. The examinations and status markings are of the same quality and produce reliable data. One limitation of this study is that the specific reason for extracting an individual tooth is often difficult to determine, as the indication for extraction may be multifactorial (e.g., a combination of deep caries, periodontal disease, and other dental conditions). Furthermore, the reported dental findings were not mutually exclusive, as severe conditions could concurrently in the same patient or even within the same tooth. Additionally, because the analyses were limited to univariate models, the observed associations—such as that between smoking and the need for dental extractions—reflect unadjusted relationships and should not be interpreted as evidence of independent effects.

In conclusion, there is a frequent need for dental treatment in HNC patients and cancer patients for whom antiresorptive therapy is scheduled. Multiple myeloma patients have less need for tooth extraction than patients receiving antiresorptive therapy and HNC patients. Furthermore, smoking and alcohol consumption are more prevalent among HNC patients than among those receiving antiresorptive therapy.

Dental examination and treatment should be provided before oncological treatment but without causing a delay in initiation of therapy. The maintenance of adequate professional and dental self‐care is also important after the initiation of oncological treatment. Future studies should elucidate whether the current focus treatment guidelines are appropriately defined to ensure sufficient oral care—but not overtreatment—prior to cancer treatment.

## Author Contributions

**Hanna Välimaa:** writing – review and editing. **Tero Puolakkainen:** writing – review and editing, formal analysis. **Aleksi Haapanen:** conceptualization, writing – review and editing, resources, methodology, investigation, funding acquisition, writing – original draft. **Johanna Uittamo:** conceptualization, writing – review and editing, methodology, writing – original draft, investigation. **Jemina Järvinen:** writing – review and editing, writing – original draft, investigation, resources, methodology, conceptualization, data curation.

## Conflicts of Interest

The authors declare no conflicts of interest.

## Data Availability

Research data are not shared.
